# Bioinformatic analysis identifies the immunological profile of turner syndrome with different X chromosome origins

**DOI:** 10.3389/fendo.2023.1024244

**Published:** 2023-01-17

**Authors:** Xiao Qi, Qinghua Wang, Mingdong Yu, Yujia Kong, Fuyan Shi, Suzhen Wang

**Affiliations:** ^1^ Department of Health Statistics, Key Laboratory of Medicine and Health of Shandong Province, School of Public Health, Weifang Medical University, Weifang, Shandong, China; ^2^ Department of Spine Surgery, Weifang People’s Hospital, Weifang, Shandong, China

**Keywords:** turner syndrome, immunological profile, gene expression omnibus, immune-related genes, protein-protein interaction, CIBERSORT, tissue-specific gene expression, WGCNA

## Abstract

**Introduction:**

Turner syndrome (TS) is a chromosomal disorder that affects phenotypic females who have one intact X chromosome and complete or partial absence of the second sex chromosome in association with one or more clinical manifestations. However, the immunological profile of TS with different X chromosome origins is incompletely understood.

**Methods:**

In this study, transcriptomic expression profiles of 26 TS (45,X) samples and 10 normal karyotype (46,XX) samples derived from GSE46687 cohort were employed. Differentially expressed immune-related genes (DEIRGs) between monosomy X TS patients with different X chromosome origins and normal females were investigated respectively. Subsequently, functional annotation, protein-protein interaction (PPI) network analysis, immunocyte infiltration evaluation, tissue-specific gene expression and Weighted gene co expression network analysis (WGCNA) were performed to explore the immunological characteristic in TS with different X chromosome origins.

**Results:**

34 and 52 DEIRGs were respectively identified in 45,Xm and 45,Xp patients compared with normal individuals. The identified DEIRGs in Xm group were significantly enriched in pathways associated with cancer. In Xp TS patients, the most enriched signals were immune response-related. A majority of genes involved in the above pathways were downregulated. PPI analysis identified 4 (*FLT3, IL3RA, CSF2RA, PIK3R3*) and 6 (*PDGFRB*, *CSF2*, *IL5*, *PRL*, *CCL17* and *IL2*)hub genes for Xm and Xp groups, respectively. CIBERSORT results showed that the proportion of Tregs in the Xm group and the naive B cells and resting NK cells in the Xp group significantly increased, respectively. Tissue-specific expression results indicated that BDCA4+_dentritic cells and CD19+ B cells were the prominent specific expressed tissues in Xp patients. Results of WGCNA support the above analysis.

**Conclusions:**

This study aims at studying the immunological characteristics of TS with different X chromosome origins. Pathways in cancer in Xm group and immune response in Xp group were suppressed. 4 and 6 hub IRGs were identified as biomarkers for Xm and Xp patients, respectively. B cells played important roles in Xp patients. Further studies are needed to draw more attention to the functional validation of these hub genes and the roles of B cells.

## Introduction

Turner syndrome (TS) is a chromosomal disorder that affects phenotypic females who have one intact X chromosome and complete or partial absence of the second sex chromosome in association with one or more clinical manifestations (1, 2). Despite the paucity of studies from Africa, Asia, and South America, Turner syndrome occurs in approximately 50 per 100000 liveborn girls in diverse ethnic populations (3). Women with Turner syndrome can exhibit different karyotypes. Typically, nearly half of women with Turner syndrome present with the 45,X karyotype (2, 4), 15–25% have mosaicism (45,X/46,XX), and the rest have X chromosomal abnormalities (5). It is characterized by infertility (6), short stature (7, 8), endocrine and metabolic disorders (9, 10), cardiovascular diseases (11), a wide range of autoimmune diseases (ADs) (12), Kawasaki disease (13), Lymphedema of hands and feet (8), vitiligo (14) and otitis media (15) et al. Many of them are related to the immune system and in general, women with 45,X karyotype are affected more than other karyotypic groups (3).

Many immune-related disorders in patients with Turner syndrome have been studied and reported. For example, the risk of ADs in TS patients is about twice that of the general female population and four times that of men (16, 17). Other disorders including celiac disease, skin disorders, rheumatic diseases, Addison disease, and immune thrombocytopenic purpura have also been reported (17). In addition, a study also detected autoantibodies (against gliadin, transglutaminase, adrenal cortex, intrinsic factors, thyroid peroxidase, and so on) in 58% of Turner syndrome women (18). In another study, the prevalence of antiGAD-65 antibody in diabetes in TS was slightly higher than that of the general population (4%vs.1.1%) (19). Nevertheless, a recent study reported that Many women with Turner syndrome lack protective antibodies to common respiratory pathogens, Haemophilus influenzae Type B and Streptococcus Pneumoniae (20).

Although many immune-related diseases and other immunological abnormalities have been reported in TS patients, the genetic basis for the predisposition is not clear (2, 12, 18). Moreover, in 45X Turner syndrome, genes may be expressed differently depending on the X chromosome origin. It has been proposed that the parental origin of the lacking X chromosome may influence the TS phenotype (21). Therefore, further studies are needed to elucidate the relationship between TS and the immune system, especially in the predominant 45X patients with maternal or paternal inherited X chromosome. So, in this study, we used statistical analysis and data mining techniques to explore the differences between monosomy X TS patients of different parental origin and normal women individuals from the immune genes, immune cells, and specific tissue localization, respectively. Here, the peripheral blood mononuclear cell (PBMC) microarray data set GSE46687 created by Bondy et al. was served as our initial data source. Our research results will help to understand the immune-related causes of TS, and provide new insights into clinical diagnosis, adequate follow-up with early detection of complications and best therapeutic intervention for immune related diseases of TS.

## Materials and methods

### Sample collection

Gene expression profiles of GSE46687 were downloaded from the Gene Expression Omnibus (GEO, https://www.ncbi.nlm.nih.gov/geo/), which was deposited by Bondy et al. The dataset was based on the GPL570 platform (HG-U133 Plus 2), containing 16 TS subjects identified as having a maternally inherited X chromosome (45, Xm), 10 TS subjects with a paternally inherited X chromosome (45, Xp), and 10 normal female karyotype subjects (46, XX). The annotation file for GPL570 was also obtained from GEO. Further, the probe expression matrix was converted to gene symbols by matching the two files. If one gene corresponded to multiple probes, the average expression value was taken for further analysis.

### Immune-related gene extraction and data processing

Immune-related genes (IRGs) were retrieved from the ImmPort database (https://www.immport.org/shared/genelists). After duplicates were removed, 1793 IRGs remained. Then, the remaining IRGs were intersected with the above preprocessed GEO dataset. The overlapped immune-related genes were selected and were transformed to log2 (expression). Finally, the gene matrix with row names as gene symbols and column names as sample names were obtained for subsequent analyses.

### Identification of DEIRGs

Differential expression analysis of immune-related genes (DEIRGs) was performed using the limma package of R software. We used t value as statistic to compare the expression of IRGs between normal 46,XX females and patients with monosomy X TS. Analyses were independently conducted for the 45,Xm and 45,Xp TS samples. P < 0.05 and |log FC| > 1 were set as the cut-off values. The results of the analysis were displayed by heatmap and volcano plot drawn in RStudio software (version: 4.1.1).

### Functional enrichment analysis of DEIRGs

DAVID 6.8 (https://david.ncifcrf.gov/tools.jsp) was used to perform the functional enrichment analysis of DEIRGs, which includes Gene Ontology (GO) terms and Kyoto Encyclopedia of Genes and Genomes (KEGG) pathways. The GO analysis described the biological domains from three aspects: biological process (BP), cellular component (CC), and molecular function (MF). KEGG pathway analysis was used to assign a series of DEIRGs to specific pathways to construct molecular interaction, reaction, and relationship networks (22). *P <* 0.05 was considered as the cut-off criteria.

### Protein-protein interaction network construction and significant modules analysis

In order to explore the interrelationship between proteins encoded by different genes, DEIRGs were imported into the STRING website (version: 11.5) for further analysis. The interaction score should be at least greater than 0.4 and isolated nodes in the network were removed. Then, we exported the analysis results into TSV format files and performed visualization and module analysis using Cytoscape software (version 3.7.1). To detect the most significant modules of hub genes from the PPI network, MCODE, a plug-in downloaded from the Cytoscape application store, was used to find tightly connected nodes in complex topology-based networks using default parameters. After that, ClueGO and CluePedia plug-ins were used to conduct and visualize the functional annotations of the selected hub genes.

### Immune cells assessment

In this study, the CIBERSORT algorithm was used to estimate the relative proportion of 22 immune cell subtypes based on gene expression. CIBERSORT is considered better than previous deconvolution methods when analyzing unknown mixture components and noise. The algorithm was set at 1000. The analysis results were filtered based on a p-value less than 0.05. Wilcoxon test was used to compare the differences in immune cell subtypes in 45,Xm and 45,Xp TS samples vs normal 46,XX females separately. The specific results were shown in a boxplot.

### Tissue-specific gene expression analysis

We analyzed the tissue-specific expression of DEIRGs using the online resource BioGPS (http://biogps.org). One gene was considered to be tissue-specific if it corresponded to a single organ or tissue with an expression value greater than 10 times the median and there was no irrelevant tissue whose expression value was greater than one-third of the maximum expression value.

### Weighted gene co-expression network analysis

Weighted gene co-expression network analysis (WGCNA) is a method to analyze the gene expression patterns of multiple samples. It can be used to find clusters (modules) of highly related genes with similar gene expression patterns and analyze the relationship between modules and specific features (23) (e.g., clinical information of patients).

Firstly, we clustered the samples and eliminated the outliers. Then the R package termed “WGCNA” was used to construct a gene co-expression network and to detect the modules. To achieve this goal, firstly, the pickSoftThreshold function performed the analysis of network topology and the appropriate soft threshold was selected on the condition that the scale-free topology fitting index (R^2^) is greater than 0.9 and the network has good average connectivity.

Then, we constructed an adjacency matrix to describe the strength of the association between the nodes. Subsequently, a topological overlap matrix (TOM) was transformed from the adjacency matrix, which was used to quantitatively describe the similarity between two nodes by comparing the weighted correlation between them and other nodes. Next, we carried out hierarchical clustering to identify modules and similar gene expressions were divided into different modules,each containing at least 30 genes (minModuleSize = 30). Finally, the expression profiles of each module were summarized by the module eigengene (ME) and the correlation between the ME and clinical features was calculated. Then according to the correlation coefficients r and the P value, the modules that most significantly associated with the clinical features were focused and the genes in these modules were selected for KEGG pathway enrichment analysis used a Cytoscape plug-in,ClueGO v2.5.9 and Cluepedia v1.5.9.

At last, we merged the DEIRGs in the Xp and Xm group with the genes in the modules, respectively, to see if all the genes in the modules expressed equally in all patients.

## Results

### Differentially expressed immune-related genes

**Figure 1:**
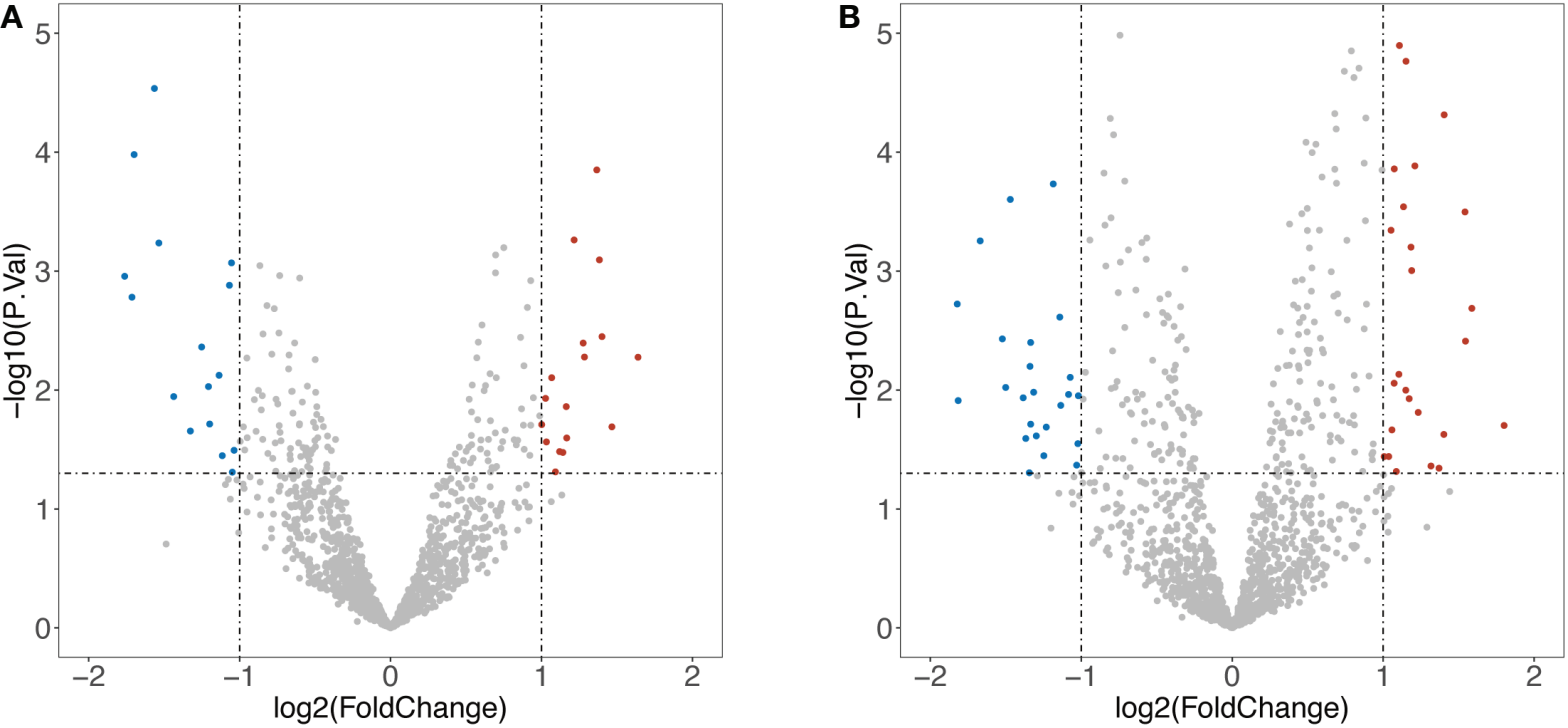
Volcano map of differentially expressed Immune-Related genes (DEIRGs). **(A)** DEIRGs between Xm TS patients and normal individuals. **(B)** DEIRGs between Xp TS patients and normal individuals.

**Figure 2:**
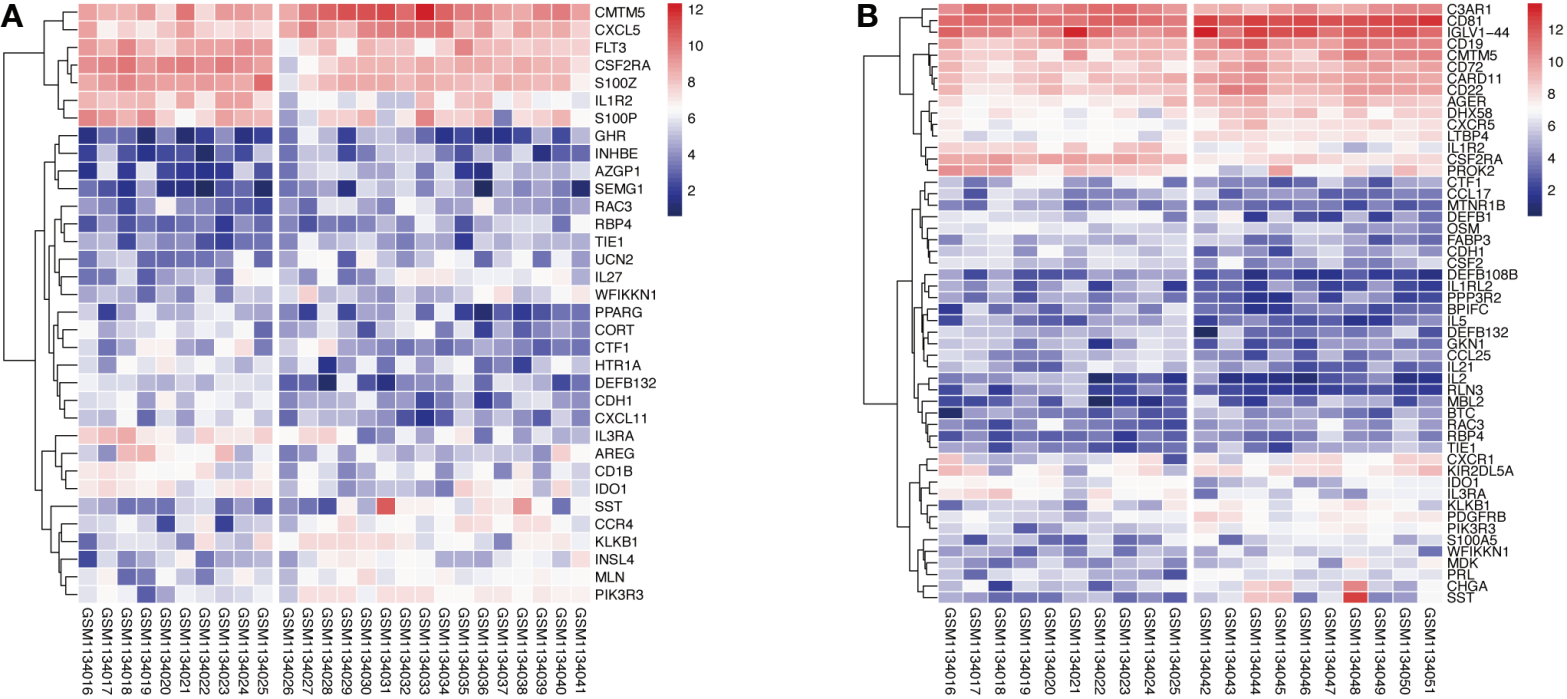
Heatmap of upregulated and downregulated genes in monosomy X TS patients compared with normal individuals. **(A)** Xm TS patients and normal individuals. The left side of the gap is the expression of the normal individuals, the right side is the Xm patients. **(B)** Xp TS patients and normal individuals. The left and right of the gap are normal individuals and Xp patients respectively.

1258 overlapped IRGs were identified after intersecting all IRGs from the ImmPort database with the GEO dataset. By using limma package to analyze the differential expression of the IRGs (DEIRGs), 34 DEIRGs were obtained composed of 18 upregulated and 16 downregulated genes between Xm TS patients and normal individuals (**Table S1**). In addition, 27 upregulated and 25 downregulated genes were identified between Xp TS patients and normal individuals (**Table S2**). Among these DEIRGs, *CSF2RA* and *IL3RA* were on the X chromosome, and were both downregulated in Xm and Xp TS patients. Eight of the upregulated genes (*CMTM5, KLKB1, PIK3R3, RAC3, RBP4, SST, TIE1, WFIKKN1*) were the same in Xm and Xp TS patients. Besides, 7 downregulated genes (*CDH1, CSF2RA, CTF1, DEFB132, IDO1, IL1R2, IL3RA*) were the same in both groups. The volcano map and heatmap for the DEIRGs of the two groups are presented in **Figure 1** and **Figure 2**.

### GO and KEGG enrichment analysis of DEIRGs

**Figure 3:**
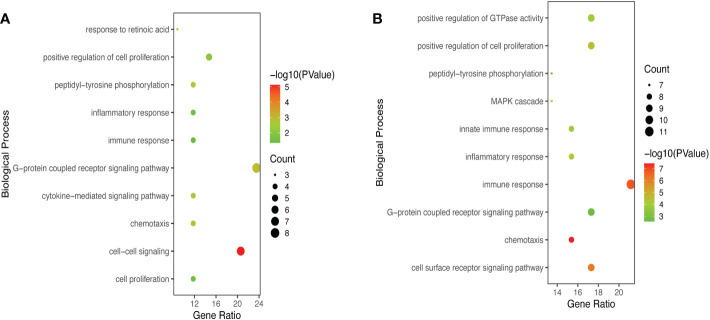
Significant enriched biological process (BP) terms between monosomy X TS patients and normal individuals. **(A)** The top 10 BP terms in DEIRGs of Xm TS patients compared with normal individuals. **(B)** The top 10 BP terms in DEIRGs of Xp TS patients compared with normal individuals.

**Figure 4:**
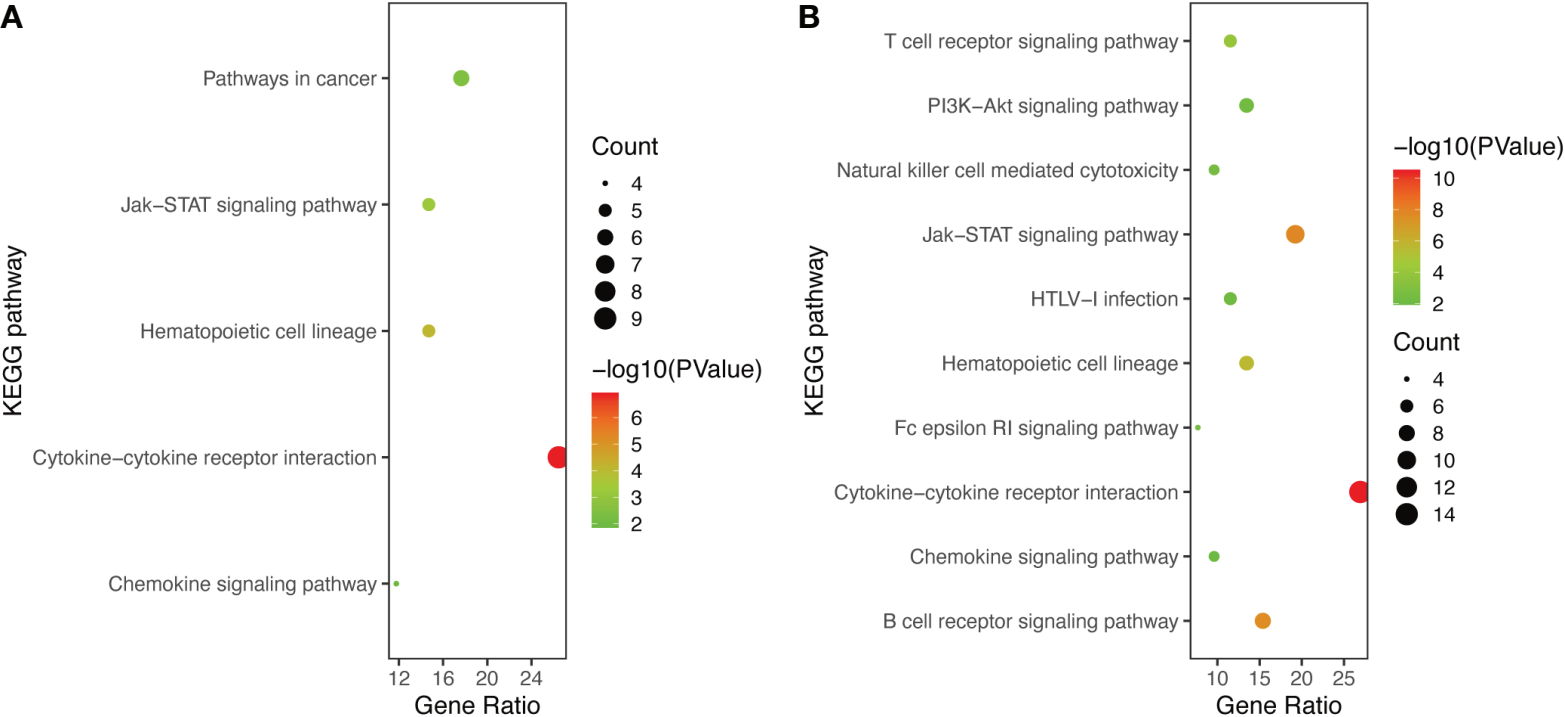
The KEGG pathways of DEIRGs. **(A)** The all 5 enriched KEGG pathways of DEIRGs between Xm TS patients and normal individuals. **(B)** The top 10 enriched KEGG pathways of DEIRGs between Xp TS patients and normal individuals.

GO and KEGG Enrichment analyses of DEIRGs were performed using the DAVID 6.8 online tool. The results were displayed by bubble graph using R package. If there were less than 10 terms or pathways, all of them were displayed; otherwise, only the top 10 were displayed (**Figures 3**, **4**, and **Figure S1**). According to the functional enrichment results, 15 BP terms were significantly enriched in DEIRGs between Xm TS patients and normal individuals. The most enriched BP terms include cell-cell signaling and G-protein coupled receptor signaling pathway (**Figure 3A**). For DEIRGs between Xp TS patients and normal individuals, there were 34 BP terms statistically significantly enriched. And the most enriched BP terms include immune response and cell surface receptor signaling pathway (**Figure 3B**). Among them, the immune response in Xp TS patients was significantly inhibited, with 8 of the 11 genes that enriched in this term downregulated, including *CCL25*, *IL21*, *CSF2*, *IL5*, *IL1R2*, *OSM*, *DEFB1*, and *IL2*. KEGG analysis results revealed that DEIRGs in Xm TS patients were involved in Cytokine-cytokine receptor interaction, Hematopoietic cell lineage, Jak-STAT signaling pathway, Pathways in cancer and Chemokine signaling pathway (**Figure 4A**). For Xp TS patients, the top 10 pathways included four pathways of the Xm group other than Pathways in cancer, and the remaining six pathways were mainly involved in immune cell-related signaling pathways (**Figure 4B**). For both groups, the most abundant pathway was the Cytokine-Cytokine receptor interaction. Moreover, most of the genes associated with the cytokine-cytokine receptor interaction pathway were downregulated, indicating that the pathway was suppressed in monosomy X TS patients.

### PPI network construction and significant module analysis of DEIRGs

**Figure 5:**
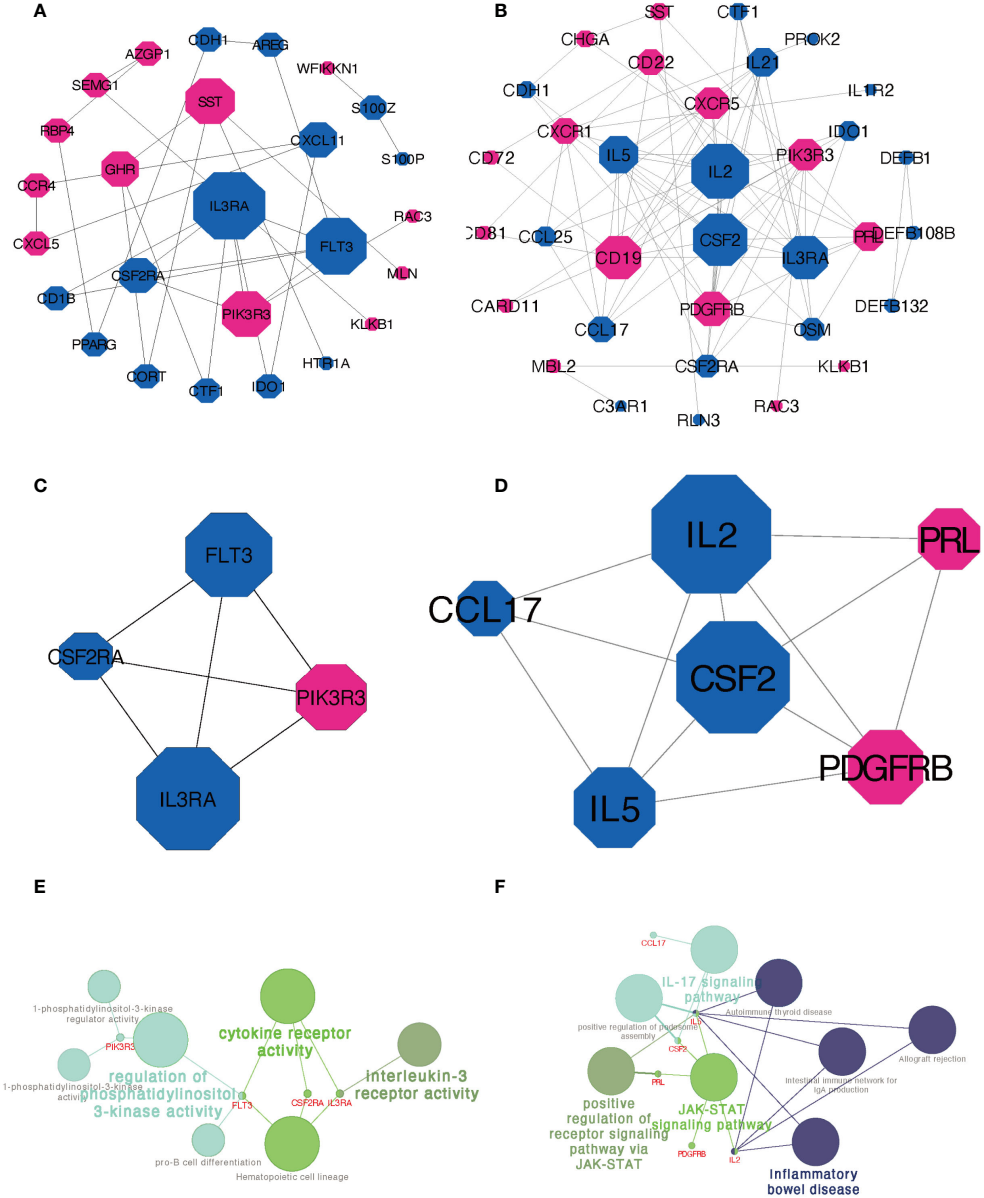
PPI network construction and module analysis. **(A)** PPI network of DEIRGs in Xm TS patients compared with normal individuals. **(B)** PPI network of DEIRGs in Xp TS patients compared with normal individuals. **(C)** The most significant module in Xm TS group. **(D)** The most significant module in Xp TS group. Upregulated genes were marked in red and downregulated genes were marked in blue. Node diameter was positively correlated with node connectivity. **(E)** Functional enrichment analysis of the hub genes in the most significant module in Xm TS group. **(F)** Functional enrichment analysis of the hub genes in the most significant module in Xp TS group.

The PPI network of DEIRGs constructed by STRING database was adjusted and visualized by Cytoscape. **Figure 5A** showed the network of DEIRGs in Xm TS patients. **Figure 5B** displayed the network in Xp TS patients. Upregulated genes were marked in red and downregulated genes were marked in blue. Node diameter was positively correlated with node connectivity. In order to find out the core modules of the complex network, we performed MCODE plug-in analysis, which identified 3 modules in both groups respectively. The most significant module with the highest score were shown in **Figures 5C, D**, which contain 4 genes with 6 edges and 6 genes with 12 edges respectively. Furthermore, functional enrichment analysis indicated that the 4 hub genes in Xm TS group were mainly involved in cytokine receptor activity, regulation of phosphatidylinositol 3-kinase activity and **i**nterleukin-3 receptor activity (**Figure 5E**). Among them, genes (*FLT3, IL3RA, CSF2RA*) involved in cytokine receptor activity were all downregulated. In Xp TS group, *CSF2* and *IL2* were determined to be the core genes according to MCODE score. The 6 hub genes were mainly involved in Inflammatory bowel disease, IL-17 signaling pathway, JAK-STAT signaling pathway, and positive regulation of receptor signaling pathway *via* JAK-STAT(**Figure 5F**). Genes involved in Inflammatory bowel disease and IL-17 signaling pathways were all downregulated.

### Immune cells assessment results

**Figure 6:**
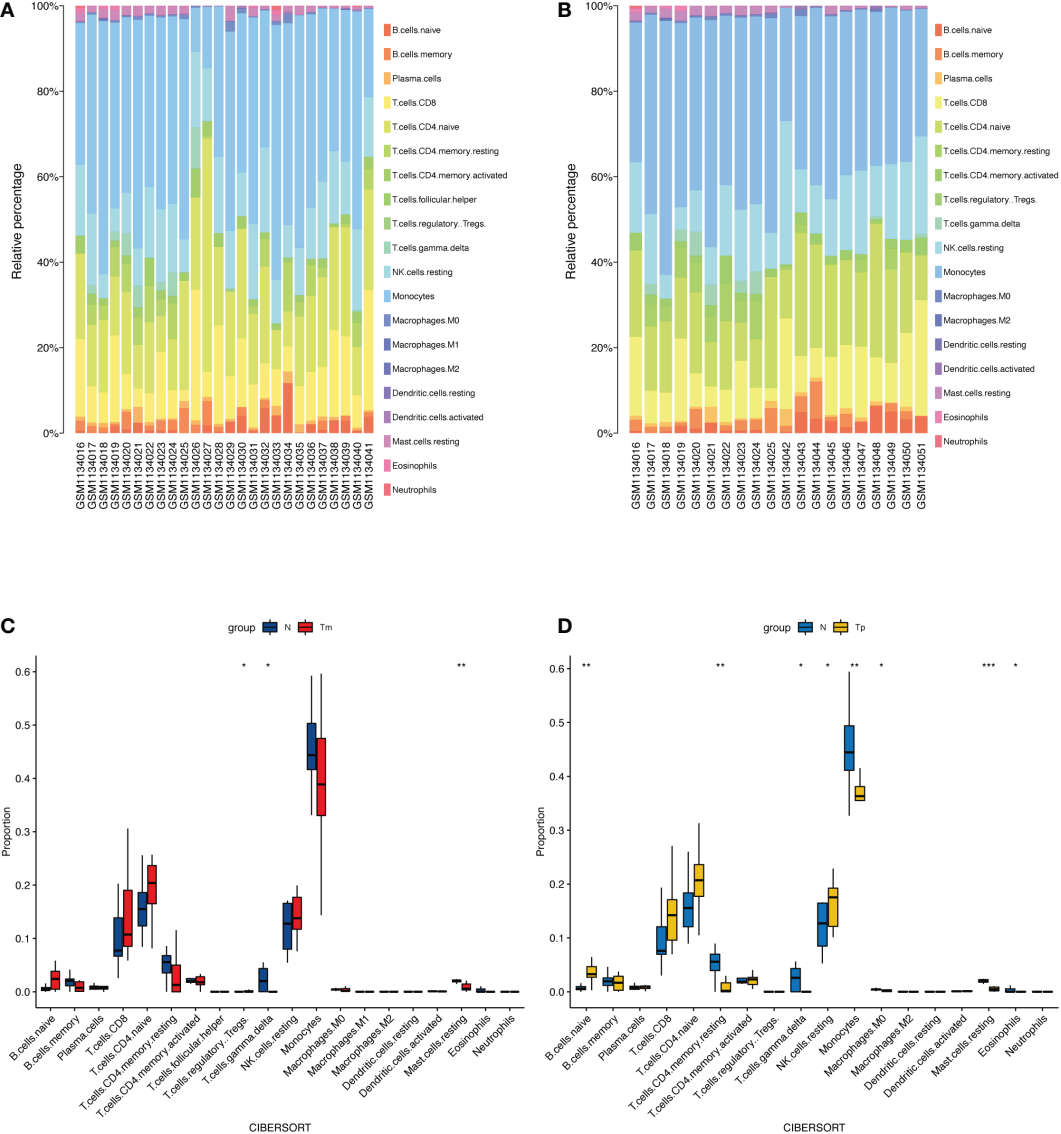
The landscape of immune infiltration between monosomy X TS patients and normal individuals. **(A)** The relative percentage of immune cells in Xm TS patients, and normal individuals. The first 10 samples were normal individuals, the rest were Xm TS patients. **(B)** The relative percentage of immune cells in Xp TS patients, and normal individuals. The first 10 samples were normal individuals, the rest were Xp TS patients. **(C)** The differences in immune cell proportions between Xm TS patients (red color) and the normal individuals (blue color). **(D)** The differences in immune cell proportions between Xp TS patients (yellow color) and the normal individuals (blue color). (*p < 0.05,** *p <* 0.01,*** *p <* 0.001).

**Figures 6A, B** shows the proportions of immune cells in monosomy X (Xm and Xp) TS patients and normal individuals calculated by the CIBERSORT algorithm. Obviously, monocytes constituted the majority of all immune cells in Xm TS patients, Xp TS patients, and the normal individuals. Meanwhile, by comparing the two images, we found that the Xp group had two fewer immune cell subpopulations than the Xm group, namely follicular helper T (TFH) cells, and M1 macrophages. Furthermore, we investigated the differences in immune cell proportions between Xm, Xp TS patients and the normal individuals, respectively. **Figure 6C** indicates that the proportions of gamma delta T cells (γδT cells) and resting mast cells in Xm TS patients were significantly decreased. However, the proportions of regulatory T cells (Tregs) were significantly increased. In **Figure 6D**, it was revealed that naive B cells and resting NK cells in Xp TS patients were significantly upregulated, whereas the proportions of resting CD4 memory T cells, gamma delta T cells (γδT cells), monocytes, M0 macrophages, and eosinophils were significantly lower than normal individuals.

### Tissue-specific expression of genes

Of the 34 differentially regulated genes in Xm TS patients, 11 were tissue-specific. Of these 11, 5 were highly expressed in the immune system, including *CD1B*, *CSF2RA*, *FLT3*, *IL1R2*, and *IL3RA*, and the 3 core genes (*CSF2RA*, *FLT3*, and *IL3RA*) mentioned above were highest expressed in BDCA4+_dentritic cells, the other two were in thymus and Bone marrow respectively. Specific results are shown in **Table 1**. In Xp TS patients, 23 of 52 differentially regulated genes were tissue-specific, excluding the two core genes, *CSF2* and *IL2*. The first 18 rows in **Table 2** show that there were 11 genes highly expressed in the immune system. Among the 11 genes, *CD19*, *CD22*, and *CD72* were highly expressed in CD19+ B cells, *CSF2RA*, *IGLV1-44*, and *IL3RA* were highest expressed in BDCA4+_dentritic cells, the other 5 were in CD14+ monocytes, Thymus, CD33+_Myeloid, Bone marrow, and Whole Blood respectively. More specific results are shown in **Table 2**.

**Table 1:** The tissue-specific genes in Xm TS patients.

Gene Symbol	Regulated condition	Gene name	Chromosome	Tissue with highest expression
CD1B	down	CD1b molecule	1	thymus
CSF2RA	down	colony stimulating factor 2 receptor subunit alpha	X	BDCA4+_dentritic cells
FLT3	down	fms related receptor tyrosine kinase 3	13	BDCA4+_dentritic cells
				CD34+
IL3RA	down	interleukin 3 receptor subunit alpha	X	BDCA4+_dentritic cells
IL1R2	down	interleukin 1 receptor type 2	2	Whole blood
				Bone marrow
INHBE	up	inhibin subunit beta E	12	Liver
INSL4	up	insulin like 4	9	Placenta
PPARG	down	peroxisome proliferator activated receptor gamma	3	Adipocyte
RBP4	up	retinol binding protein 4	10	liver
SEMG1	up	semenogelin 1	20	prostate
SST	up	somatostatin	3	Pancreatic islet

**Table 2:** The tissue-specific genes in Xp TS patients.

Gene Symbol	Regulated condition	Gene name	Chromosome	Tissue with highest expression
C3AR1	down	complement C3a receptor 1	12	CD14+ monocytes
CCL25	down	C-C motif chemokine ligand 25	19	Thymus
				Small intestine
CD19	up	CD19 molecule	16	CD19+ B cells (neg._nel)
				Lymphoma burkitts (Raji)
CD22	up	CD22 molecule	19	Lymphoma burkitts(Raji)
				CD19+ Bcells (neg._nel)
CD72	up	CD72 molecule	9	CD19+ Bcells (neg._nel)
				Lymphoma burkitts(Raji)
CSF2RA	down	colony stimulating factor 2 receptor subunit alpha	X	BDCA4+_dentritic cells
PROK2	down	prokineticin 2	3	Whole Blood
				CD33+_Myeloid
IGLV1-44	up	immunoglobulin lambda variable 1-44	22	BDCA4+_dentritic cells
				Small intestine
IL1R2	down	interleukin 1 receptor type 2	2	Whole Blood
				Bone marrow
IL3RA	down	interleukin 3 receptor subunit alpha	X	BDCA4+_dentritic cells
CXCR1	up	C-X-C motif chemokine receptor 1	2	Whole Blood
CHGA	up	chromogranin A	14	Pancreatic islet
DEFB1	down	defensin beta 1	8	Salivary gland
FABP3	down	fatty acid binding protein 3	1	Heart
LTBP4	up	latent transforming growth factor beta binding protein 4	19	Thyroid
MBL2	up	mannose binding lectin 2	10	Liver
MDK	up	midkine	11	Colorectal adenocarcinoma
PDGFRB	up	platelet derived growth factor receptor beta	5	Adipocyte
PRL	up	prolactin	6	Pituitary
RBP4	up	retinol binding protein 4	10	liver
SST	up	somatostatin	3	Pancreatic islet
AGER	up	advanced glycosylation end-product specific receptor	6	Lung
TIE1	up	tyrosine kinase with immunoglobulin like and EGF like domains 1	1	Lung

### WGCNA and key modules analysis

Co-expression analysis was carried out to construct the co-expression network. In this study, We have chosen the soft thresholding power 5 to ensure a scale-free network (**Figures S2A, B**). Then ten modules were identified based on average hierarchical clustering and dynamic tree clipping (**Figure S2C**). In order to pick out important clinical modules, the relationship between the modules and the external traits was studied. From **Figure S2D**, we notice that there are five (brown, black, magenta, red and turquoise), two (purple and red) and four (brown, magenta, red and grey) key modules significantly associate with the control(normal individuals), Xm and Xp group, respectively. Considering the correlation coefficients r, we chose the red and brown module as our target modules for the next analysis. Among them, the red module was significantly negatively correlated with the normal individuals and positively correlated with monosomy X groups, and the brown module was just the opposite.

72 genes in the red module and 102 genes in the brown module were provided as input into ClueGO for analysis, respectively. As shown in **Figure S3A**, genes in the red module are significantly enriched in five terms, in which the B cell receptor signaling pathway accounts for 91.89% (**Figure S3B**). In the brown module, pathway enrichment shows that the genes are enriched in nine pathways, with the Ras signaling pathway and the rheumatoid arthritis pathway accounting for more than 50%(**Figures S3C, D**). And 12 genes in the red module and 7 genes in the brown module coincided with the DEIRGs of the Xp group, including *PDGFRB* and *CSF2* in the 6 hub genes identified by MCODE earlier (**Table S3**). At the same time, 6 genes in the red module and 8 genes in the brown module were consistent with the DEIRGs of the Xm group, containing all four hub genes (*FLT3*, *IL3RA*, *CSF2RA*, and *PIK3R3*) determined by MCODE (**Table S3**).

## Discussion

In this study, we aimed to identify the immunological profile of turner syndrome with different X chromosome origins from the immune genes, immune cells, and specific tissue localization, respectively. First, we analyzed the DEIRGs for the 45,Xm and 45,Xp TS samples independently with the nomal individuals to determine whether there was any difference between monosomy X TS patients of different parent origin. As a result, 18 upregulated and 16 downregulated DEIRGs were screened out between Xm TS patients and normal individuals, and 27 upregulated and 25 downregulated genes were identified between Xp TS patients and normal individuals. Obviously, more DEIRGs were present if lacking maternal origin X chromosome.

Then, DEIRGs in both groups were annotated by functional enrichment analysis separately. According to the results, DEIRGs in Xp group were mainly enriched in immune response and immune-related signaling pathways. 8 of the 11 genes that enriched in immune response were downregulated, indicating that the immune response was significantly inhibited in Xp TS patients. This result was to some extent consistent with the findings of Swee DS (20), which displayed that 69% of 114 consecutive adults with TS reported low baseline Antibody levels, and 20% of these patients reported persistent low antibody levels after immunization. KEGG analysis results revealed that DEIRGs in Xm TS patients were significantly involved in Pathways in cancer. There were 6 genes enriched in the pathway, among which 4 genes were downregulated, indicating that in Xm TS patients Pathways in cancer were suppressed. Some epidemiological studies have found that the overall risk of cancer in patients with Turner syndrome does not increase and even the incidence of breast cancer is much lower than in women of 46,XX (24–26). This study explains these results at the genetic level.

The PPI network and key module analyses identified 4 and 6 hub genes for Xm and Xp groups separately. *FLT3*, *IL3RA*, *CSF2RA*, and *PIK3R3* figured prominently in the PPI network of Xm group. *IL3RA* (interleukin 3 receptor subunit alpha) and *CSF2RA* (colony stimulating factor 2 receptor subunit alpha), residing in pseudoautosomal region of the human sex chromosomes, were differentially methylated among females with TS compared with controls (27). *IL3RA* was speculated to be linked to the increased risk of autoimmune diseases in Turner syndrome in many studies (1). The protein encoded by *CSF2RA* is a cytokine, which controls the production, differentiation, and functions of macrophages and granulocytes (28). In addition, *CSF2RA*, previously considered to be associated with early intrauterine death, might be associated with a significantly increased risk of spontaneous abortion in fetuses with a 45,X chromosome karyotype (29). FLT3, FMS-like tyrosine kinase 3, a receptor tyrosine kinase, plays an important role in regulating immunity because of its importance in the development of B cell lineage and dendritic cells (30). PIK3R3(phosphoinositide-3-kinase regulatory subunit 3)has been reported to increase in Inflammatory bowel disease (IBD) patients, which indicates that PIK3R3 could be a therapeutic target for IBD (31).

In Xp TS group, the 6 hub genes were mainly enriched in Inflammatory bowel disease, IL-17 signaling pathway, and JAK-STAT signaling pathway. Genes involved in Inflammatory bowel disease and IL-17 signaling pathways were all downregulated. The combined relative risk of IBD (ulcerative colitis and Crohn’s disease) was 2.25(0.61-5.75) compared with 46, XX women (17, 32). IL-17 is a founding member of a new family of inflammatory cytokines (33). Uncontrolled acceleration of the system or brake failure can lead to persistent inflammation, which can lead to tissue damage (33). The JAK-STAT signaling pathway is involved in the pathogenesis of inflammation and autoimmune diseases, including rheumatoid arthritis, psoriasis, and inflammatory bowel disease (34). The success of small-molecule JAK inhibitors (Jakinibs) in the treatment of rheumatism suggests that intracellular signaling pathways can be targeted to the treatment of immune related diseases (35, 36).

CIBERSORT algorithm was used to assess the proportion of immune cells in TS patients. In Xm group, the proportions of gamma delta T cells (γδ T cells) and resting mast cells were significantly decreased, whereas regulatory T cells (Tregs) were significantly increased. In Xp group, naive B cells and resting NK cells were significantly upregulated, whereas the proportions of resting CD4 memory T cells, gamma delta T cells (γδT cells), resting mast cells, monocytes, M0 macrophages, and eosinophils were significantly lower than normal individuals.

There were not many studies on immune cell subsets in Turner syndrome and the results were inconsistent, especially the results about Tregs. One research reported that CD16 NK cells increased in TS (37), which was consistent with our research in Xp group to some extent. Yet another study did not find any significant differences in the proportion of B cells, macrophages, or NK cells in TS patients (38), which was different from our results. But a low percentage of CD4 was reported in several studies (16, 37, 39), just as in our Xp group. Regulatory T (Treg) cells are very important for peripheral immune tolerance and the prevention of autoimmune and tissue damage (40). Therapeutic administration of Tregs (experimental phase) to prevent the development of type 1 diabetes is being tested (41). However, the research results about Tregs were inconsistent. Gawlik AM et al. (16) reported no difference in the percentage of Tregs between TS patients and the controls. Sznurkowska et al. (38) found that the Tregs% was higher in children with newly diagnosed juvenile idiopathic arthritis (JIA) without treatment than in controls. And in our study, Tregs% in Xm TS group was significantly increased compared with normal individuals. Studies have also pointed out that GH treatment for TS patient does not significantly change the patient’s immune function (37). In view of the inconsistency of research results, the significance of immune cell subsets in TS needs more research to confirm.

Tissue-specific expression analysis revealed that the highest specific system in terms of DEIRGs expression was the immune system in both groups. In particular, 3 core genes(*CSF2RA*, *FLT3*, and *IL3RA*) identified by MCODE of Xm TS patients and 3 genes (*CSF2RA*, *IGLV1-44*, and *IL3RA*) in Xp group specifically expressed in BDCA4+_dentritic cells. Dendritic cells (DC) is the only antigen-presenting cell that can trigger naive Th cells and initiate an immune response. DC are considered to be the key mediators of immune tolerance (42). BDCA4+_dentritic cells specific expressed genes dysregulation may result in the alteration of immune tolerance. In Xp group, there were 3 genes (*CD19*, *CD22*, and *CD72*) specifically expressed in CD19+ B cells. Studies have shown that compared with the healthy control, the expression of CD19+ B cells in peripheral blood of SLE was significantly higher (43, 44), which indicated that the CD19+ B cells may play important roles in immune related diseases.

In the results of WGCNA, 91.89% genes in the red module, which was significantly positively correlated with monosomy X groups, were enriched in the B cell receptor signaling pathway. This result supported the results from the immunocyte infiltration evaluation and tissue-specific gene expression analysis. And over 50% genes in the brown module that negatively correlated with the Xp group were enriched in the Ras signaling pathway and the rheumatoid arthritis pathway. The result was complementary to the results of functional annotation, protein-protein interaction (PPI) network analysis. The results that the red and brown module contained the hub genes of the Xm and Xp group provided poof of concept and identified preliminary possible targets in turner patients.

This study had several limitations. First, our study needs a larger sample size and more data sets to valid the results in future studies. Second, since our research data was from a public dataset, there was no information on age and health status as well as individual drug use et al. Third, a major limitation of the study is that the type of study design is cross-sectional rather than cohort. Fourth, although a peripheral blood karyotype is usually adequate, a second tissue is needed to study the gene expression on the phenotype in 45,X. Fifth, further studies are needed to establish the impact of DNA-methylation and detect the differential-exon-usage from RNA-seq data.

In conclusion, this study aims at studying the immunological characteristics of TS with different X chromosome origins. The Xp TS patients showed more variability in immune genes, immune cells, and tissue specificity than that of Xm TS patients. Pathways in cancer in Xm group and immune response in Xp group were suppressed. 4 and 6 hub IRGs were identified as biomarkers for Xm and Xp patients respectively. B cells played important roles in Xp patients. Further studies are needed to draw more attention to the functional validation of these hub genes and B cells.

## Data availability statement

The original contributions presented in the study are included in the article/**Supplementary Material**. Further inquiries can be directed to the corresponding authors.

## Author contributions

FS and SW conceived and designed the experiments. YK and MY sorted out the data. XQ performed the data analysis. XQ and QW contributed to the writing and revising of this manuscript. QW and SW supervised this study and revised the manuscript. All authors contributed to the article and approved the submitted version.
