# Supplementary material for: Bioinformatic analysis identifies the immunological profile of turner syndrome with different X chromosome origins
Source: Front Endocrinol (Lausanne). 2023 Jan 17;14:1024244. doi: 10.3389/fendo.2023.1024244 (PMC9887020; doi:10.3389/fendo.2023.1024244)
Supplement: Supplementary file 2 [file DataSheet_2.pdf]

Table S1: Differentially expressed immune-related genes (DEIRGs) between Xm TS patients and normal individuals

Gene.Symbol	Name	Chromosome	logFC	P.Value
Up regulated genes				
AZGP1	alpha-2-glycoprotein 1, zinc-binding	7	1.466220543	0.020388271
CCR4	C-C motif chemokine receptor 4	3	1.400561095	0.003554784
CMTM5	CKLF like MARVEL transmembrane domain containing 5	14	1.383406063	0.000804173
CXCL5	C-X-C motif chemokine ligand 5	4	1.215979302	0.000546918
GHR	growth hormone receptor	5	1.092357569	0.048749747
IL27	interleukin 27	16	1.166991148	0.025293412
INHBE	inhibin subunit beta E	12	1.141435894	0.033468306
INSL4	insulin like 4	9	1.284553086	0.005278449
KLKB1	kallikrein B1	4	1.163817384	0.013787769
MLN	motilin	6	1.067283789	0.007873568
PIK3R3	phosphoinositide-3-kinase regulatory subunit 3	1	1.366165379	0.000140954
RAC3	Rac family small GTPase 3	17	1.032575405	0.027289601
RBP4	retinol binding protein 4	10	1.275950935	0.004026117
SEMG1	semenogelin 1	20	1.639372034	0.005296096
SST	somatostatin	3	2.051227303	0.005539639
TIE1	tyrosine kinase with immunoglobulin like and EGF like domains 1	1	1.026743571	0.011748725
UCN2	urocortin 2	3	1.119712791	0.032922125
WFIKKN1	WAP, follistatin/kazal, immunoglobulin, kunitz and netrin domain containing 1	16	1.00133285	0.019527038
Down regulated genes				
AREG	amphiregulin	4	-1.198299967	0.019307956
CD1B	CD1b molecule	1	-1.136063235	0.00751384
CDH1	cadherin 1	16	-1.534847518	0.000580232
CORT	cortistatin	1	-1.036547989	0.032112354
CSF2RA	colony stimulating factor 2 receptor subunit alpha	X	-1.564323694	2.91E-05

CTF1	cardiotrophin 1	16	-1.048159186	0.049034805
CXCL11	C-X-C motif chemokine ligand 11	4	-1.206124519	0.0093521
DEFB132	defensin beta 132	20	-1.712967474	0.001656173
FLT3	fms related receptor tyrosine kinase 3	13	-1.067401388	0.001315472
HTR1A	5-hydroxytryptamine receptor 1A	5	-1.114758556	0.035639649
IDO1	indoleamine 2,3-dioxygenase 1	8	-1.251348863	0.004345571
IL1R2	interleukin 1 receptor type 2	2	-1.698613221	0.000104709
IL3RA	interleukin 3 receptor subunit alpha	X	-1.761329527	0.001105231
PPARG	peroxisome proliferator activated receptor gamma	3	-1.43649237	0.011347671
S100P	S100 calcium binding protein P	4	-1.325988142	0.022116105
S100Z	S100 calcium binding protein Z	5	-1.053566773	0.000852462

Table S2: Differentially expressed immune-related genes (DEIRGs) between Xp TS patients and normal individuals

Gene.Symbol	Name	Chromosome	logFC	P.Value
Up regulated genes				
AGER	advanced glycosylation end-product specific receptor	6	1.134704217	0.000287817
BTC	betacellulin	4	1.036738904	0.036224694
CARD11	caspase recruitment domain family member 11	7	1.107786635	1.27E-05
CD19	CD19 molecule	16	1.403997149	4.85E-05
CD22	CD22 molecule	19	1.210033691	0.000130427
CD72	CD72 molecule	9	1.052230683	0.000453192
CD81	CD81 molecule	11	1.022765527	1.72E-07
CHGA	chromogranin A	14	1.800999884	0.019855482
CMTM5	CKLF like MARVEL transmembrane domain containing 5	14	1.10407548	0.007372116
CXCR1	C-X-C motif chemokine receptor 1	2	1.316647968	0.043525997
CXCR5	C-X-C motif chemokine receptor 5	11	1.073442265	0.000138166
DHX58	DExH-box helicase 58	17	1.188991428	0.00098955
IGLV1-44	immunoglobulin lambda variable 1-44	22	1.149761516	0.010026845
KIR2DL5A	killer cell immunoglobulin like receptor, two Ig domains	19	1.086990386	0.048404351

	and long cytoplasmic tail 5A			
KLKB1	kallikrein B1	4	1.545352289	0.003881537
LTBP4	latent transforming growth factor beta binding protein 4	19	1.151243887	1.72E-05
MBL2	mannose binding lectin 2	10	1.369835686	0.045401812
MDK	midkine	11	1.587349658	0.002055223
PDGFRB	platelet derived growth factor receptor beta	5	1.184246197	0.000628497
PIK3R3	phosphoinositide-3-kinase regulatory subunit 3	1	1.542014294	0.000317951
PRL	prolactin	6	1.172191983	0.011816937
RAC3	Rac family small GTPase 3	17	1.232132174	0.015424685
RBP4	retinol binding protein 4	10	1.072549529	0.008760042
S100A5	S100 calcium binding protein A5	1	1.401976022	0.023612491
SST	somatostatin	3	2.661665411	0.007097144
TIE1	tyrosine kinase with immunoglobulin like and EGF like domains 1	1	1.006581906	0.036245107
WFIKKN1	WAP, follistatin/kazal, immunoglobulin, kunitz and netrin domain containing 1	16	1.058046009	0.021633641
Down regulated genes				
BPIFC	BPI fold containing family C	22	-1.384737495	0.011618013
C3AR1	complement C3a receptor 1	12	-1.185596897	0.000184955
CCL17	C-C motif chemokine ligand 17	16	-1.030447804	0.042815267
CCL25	C-C motif chemokine ligand 25	19	-1.135782894	0.013457239
CDH1	cadherin 1	16	-1.335311291	0.003982051
CSF2	colony stimulating factor 2	5	-1.084135309	0.010895717
CSF2RA	colony stimulating factor 2 receptor subunit alpha	X	-1.539467461	5.57E-08
CTF1	cardiotrophin 1	16	-1.297762549	0.024305391
DEFB1	defensin beta 1	8	-1.815281121	0.012263299
DEFB108B	defensin beta 108B	11	-1.669474444	0.000556863
DEFB132	defensin beta 132	20	-1.821457733	0.001890483
FABP3	fatty acid binding protein 3	1	-1.02255724	0.028231333
GKN1	gastrokine 1	2	-1.247958767	0.035676105

IDO1	indoleamine 2,3-dioxygenase 1	8	-1.073083849	0.007824042
IL1R2	interleukin 1 receptor type 2	2	-1.469991638	0.000249587
IL1RL2	interleukin 1 receptor like 2	2	-1.367556798	0.025541576
IL2	interleukin 2	4	-1.344021873	0.049599745
IL21	interleukin 21	4	-1.335112258	0.019378002
IL3RA	interleukin 3 receptor subunit alpha	X	-1.522691868	0.003713693
IL5	interleukin 5	5	-1.231879346	0.020496939
MTNR1B	melatonin receptor 1B	11	-1.019160762	0.011163798
OSM	oncostatin M	22	-1.142195368	0.002436325
PPP3R2	protein phosphatase 3 regulatory subunit B, beta	9	-1.315371804	0.010432026
PROK2	prokineticin 2	3	-1.500574274	0.009511647
RLN3	relaxin 3	19	-1.339437745	0.006323587

Table S3: Differentially expressed immune-related genes (DEIRGs) of the Xp and Xm group in red and brown modules

Module	Xp group	Xm group
Red	CSF2, DEFB1, DHX58, IL21, IL3RA, LTBP4, MDK, PIK3R3 , SST	CCR4, CD1B, CDH1, IL3RA, PIK3R3, SST
Brown	C3AR1, CARD11, CSF2RA, IDO1, IL1R2, PDGFRB, PROK2	AZGP1, CSF2RA, CXCL11, FLT3, IDO1, IL1R2, MLN, PPARG

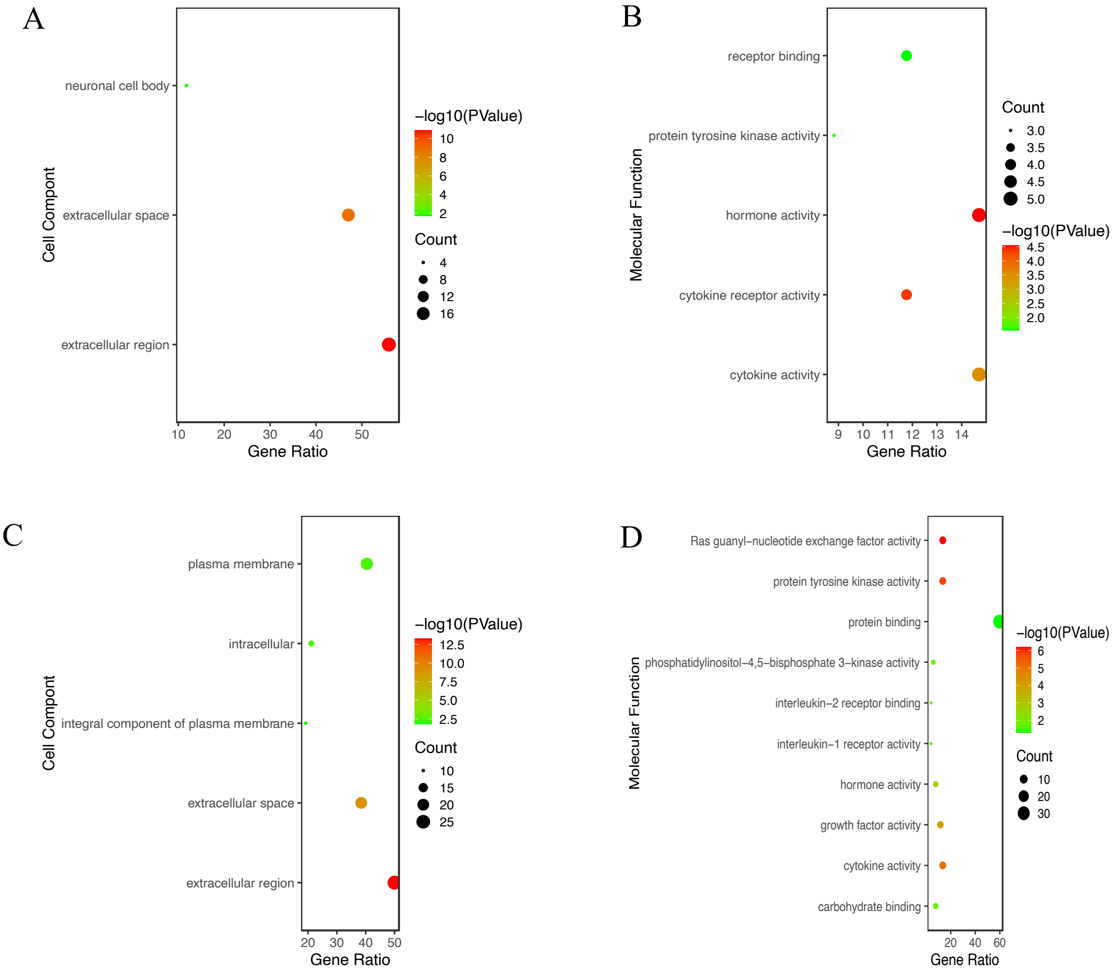

Figure S1. Significant enriched cellular component (CC) and molecular function (MF) terms between monosomy X TS patients and normal individuals. (A) The CC terms in DEIRGs of Xm TS patients compared with normal individuals. (B) The MF terms in DEIRGs of Xm TS patients compared with normal individuals. (C) The CC terms in DEIRGs of Xp TS patients compared with normal individuals. (D) The MF terms in DEIRGs of Xp TS patients compared with normal individuals.

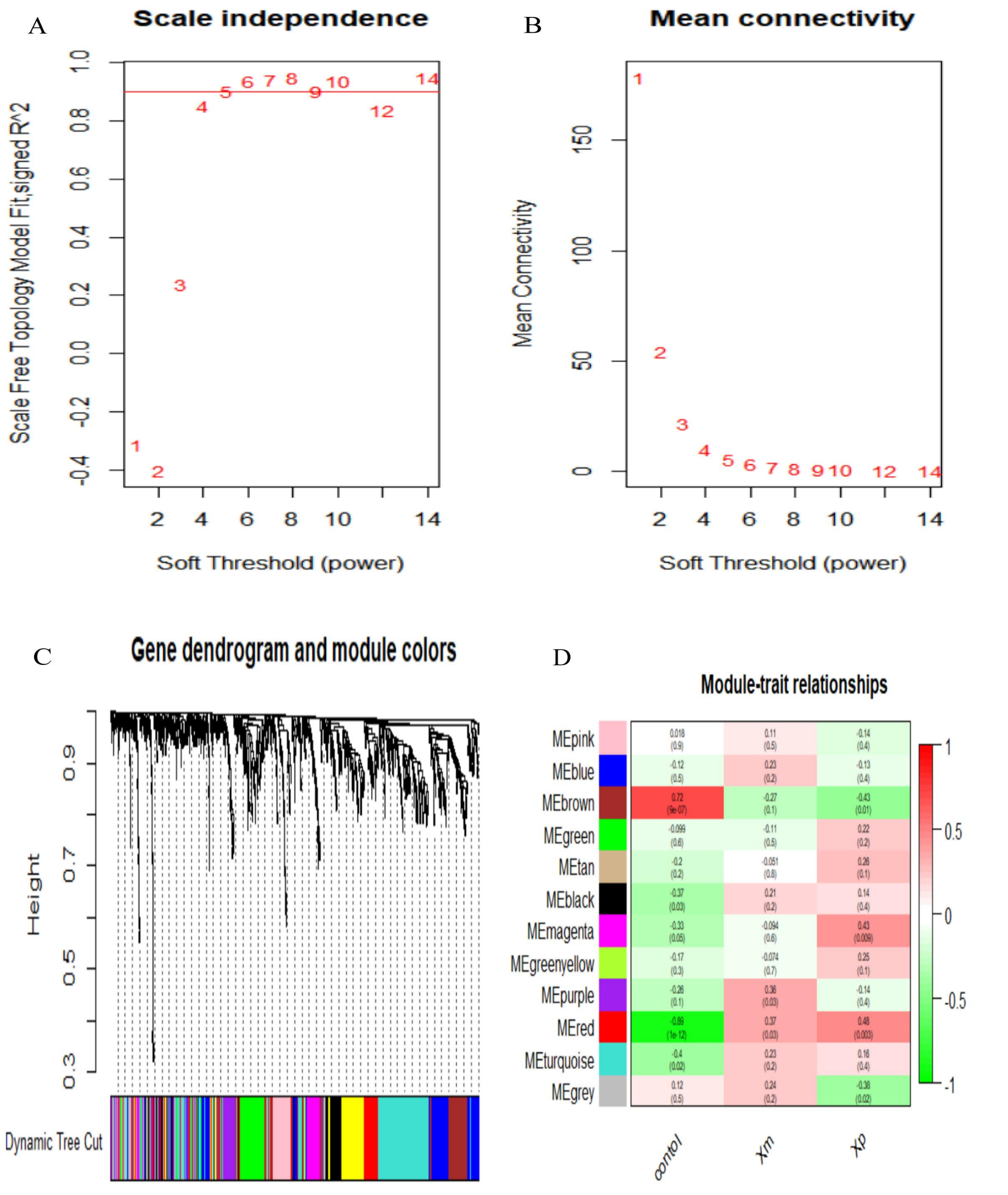

Figure S2. WGCNA analysis and Clinically Significant Module Identification. (A) Analysis of the scale-free index for various soft-threshold powers. (B) Analysis of the mean connectivity for various soft-threshold powers. (C) Clustering dendrogram of genes, with dissimilarity based on topological overlap, together with assigned module colors. (D) Module-trait associations. Each row corresponds to a module eigengene, column to a trait. Each cell contains the corresponding correlation and p-value. The table is color-coded by correlation according to the color legend.

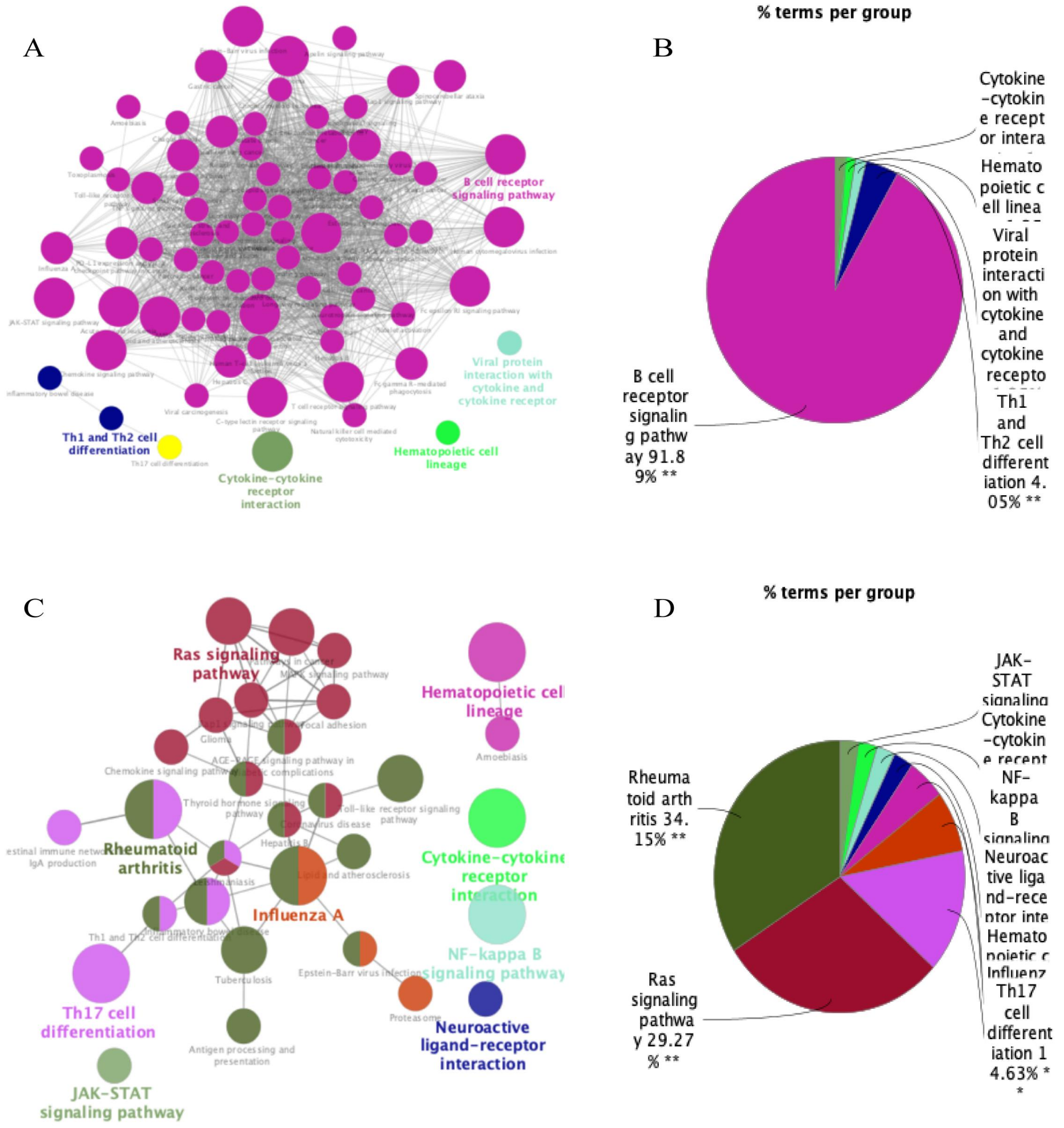

Figure S3. ClueGO enrichment analysis. (A) The interaction network of KEGG pathways for genes in the red module generated by the Cytoscape plug-in ClueGO. The most significant term of each group is highlighted. (B) Proportion of each KEGG pathways group in the total in the red module. (C) The interaction network of KEGG pathways for genes in the brown module generated by the Cytoscape plug-in ClueGO. The most significant term of each group is highlighted. (D) Proportion of each KEGG pathways group in the total in the brown module. KEGG, Kyoto Encyclopedia of Genes and Genomes . **$p < 0.05$.
